# Autoimmune thyroid disease in inborn errors of immunity: a retrospective perspective

**DOI:** 10.1186/s13023-025-04085-5

**Published:** 2025-10-23

**Authors:** Makbule Seda Bayrak Durmaz, Betul Ozdel Ozturk, Begum Gorgulu Akin, Fikriye Kalkan, Sadan Soyyiğit

**Affiliations:** 1 Department of Immunology and Allergic Diseases, Ankara Bilkent City Hospital, Ankara, 06800 Turkey; 2 Division of Allergy and Clinical Immunology, Ankara Bilkent City Hospital, School of Medicine, Ankara Yildirim Beyazit University, Ankara, 06800 Turkey

**Keywords:** Autoimmune thyroid disease, Hashimoto’s thyroiditis, Inborn errors of immunity, Thyroid autoantibodies, Immune dysregulation

## Abstract

**Background:**

Autoimmune thyroid diseases (AITDs) are among the most frequently reported autoimmune manifestations in individuals with inborn errors of immunity (IEIs). While immune dysregulation in IEI is known to predispose patients to autoimmunity, data regarding the prevalence and characteristics of AITD in this population remain limited. This study aimed to assess the frequency of AITD and describe its clinical, serological, ultrasonographic, and immunological features in a cohort of adult IEI patients.

**Methods:**

This retrospective study included 45 adult IEI patients, excluding those with selective IgA deficiency. Demographic, clinical, and immunological data were collected from medical records. AITD diagnosis was based on thyroid-specific autoantibodies, thyroid function tests, and/or ultrasonographic features consistent with thyroiditis. Comparative analyses were conducted between patients with and without AITD.

**Results:**

AITD was identified in 7 patients (15.5%), including 6 with Hashimoto’s thyroiditis (HT) and 1 with seronegative chronic autoimmune thyroiditis (SN-CAT); no cases of Graves’ disease were observed. The majority of patients with AITD were female (85.7%), while only one case occurred in a male (14.3%). No statistically significant sex-based difference in AITD frequency was detected (*p* = 0.225). Anti-thyroid peroxidase antibodies were positive in 85.7% of AITD patients, and anti-thyroglobulin antibodies in 60.0%. All were euthyroid at evaluation, though 57.1% were receiving L-thyroxine therapy. Non-AITD thyroid abnormalities were present in 12 patients (26.6%), including isolated serology and a solitary thyroid nodule. Compared with non-AITD patients, those with AITD exhibited significantly higher CD16⁺56⁺ NK cell percentages (*p* = 0.010), while other lymphocyte subsets did not differ. No significant differences were observed regarding sex, IEI phenotype, non-thyroidal autoimmunity, or infection-related complications.

**Conclusions:**

In this adult IEI cohort, HT was the most prevalent autoimmune thyroid condition. Distinct immunological features, particularly increased NK cell frequencies, may reflect underlying immune dysregulation. These findings support systematic thyroid evaluation, even in asymptomatic IEI patients, and emphasize the need for larger, genetically diverse cohorts to validate and expand these observations.

## Introduction

Autoimmune thyroid diseases (AITDs), including Hashimoto’s thyroiditis (HT) and Graves’ disease (GD), are among the most common organ-specific autoimmune disorders in the general population [1–3]. Both subtypes are characterized by lymphocytic infiltration of the thyroid gland but differ in clinical presentation: HT most often leads to hypothyroidism, but may also present as euthyroidism or, less commonly, a transient hyperthyroid phase (hashitoxicosis), and is frequently associated with progressive fibrosis [4]. GD, by conrast, is typically associated with hyperthyroidism mediated by thyroid-stimulating immunoglobulins. These disorders involve T-cell-mediated immune dysregulation, B-cell activation, and the production of thyroid-specific autoantibodies, such as anti-thyroid peroxidase (anti-TPO), anti-thyroglobulin (anti-Tg), and thyroid-stimulating hormone receptor antibodies (TRAb) [1–3, 5]. Although these autoantibodies are useful diagnostic markers, they may be absent in approximately 5–10% of patients [4]. In such cases, characteristic thyroid ultrasonographic (USG) features, such as diffuse hypoechogenicity and heterogeneity, can support the diagnosis of autoimmune thyroiditis. The term ‘seronegative chronic autoimmune thyroiditis’ (SN-CAT) refers to patients with these typical sonographic features in the absence of detectable thyroid autoantibodies; this condition may present with overt or subclinical hypothyroidism, or, less frequently, with preserved euthyroid function [6–8].

Although genetic, hormonal, and environmental factors contribute to disease development, impaired immune tolerance is considered a central mechanism [1–3, 5, 9]. Thyroid hormones play a crucial role in metabolism, growth, development, and immune regulation [10]. Additionally, emerging evidence has linked thyroid dysfunction to certain neuropsychiatric disorders [11]. Given this broad systemic impact, early identification and monitoring of thyroid dysfunction are important for improving clinical outcomes. While AITDs are well-described in the general population, their diagnosis in **inborn errors of immunity (**IEIs) patients may be more challenging due to atypical or subclinical presentations [12, 13].

IEIs are a heterogeneous group of disorders that affect innate and adaptive immune pathways. While classically linked to recurrent infections, IEIs are now increasingly recognized for their association with immune dysregulation, including autoimmunity, allergy, and malignancy [14, 15]. Autoimmune diseases represent one of the most frequent non-infectious manifestations in IEI, with autoimmune endocrine disorders, particularly AITD, being commonly reported [10, 16–19]. However, humoral immune defects in IEI may impair antibody production, limiting the sensitivity of conventional serological tests [14, 20]. In addition, immunoglobulin replacement therapy (IGRT) may lead to passive transfer of donor-derived antibodies, potentially masking a patient’s true serological status [21]. Consequently, a comprehensive evaluation—including thyroid function tests and USG—is suggested to aid diagnosis in this patient population.

Although previous reports have noted autoimmune endocrinopathies in IEI, data specifically characterizing AITD in these patients remain limited. In this retrospective study, we aimed to assess the frequency of AITD and to describe its clinical, serological, USG, and immunological features in a cohort of adult IEI patients. We also sought to compare these findings with classical AITD profiles described in the general population, to highlight potential distinctions compared to classical AITD presentations.

## Materials and methods

### Study design and ethics approval

This retrospective observational study was conducted at the Adult Immunology Clinic of Ankara Bilkent City Hospital and approved by the Local Ethics Committee (Approval No: TABED 1-25-1417), in accordance with the principles of the Declaration of Helsinki.

### Patient selection

Patients aged 18 years or older who were regularly followed in our clinic between 2019 and 2025 and had a confirmed diagnosis of IEI based on the criteria of the European Society for Immunodeficiencies (ESID) were eligible for inclusion [22]. Only patients with complete clinical and laboratory records were included. Individuals with selective or partial IgA deficiency were excluded due to their high prevalence and distinct clinical characteristics. Additional exclusion criteria were age under 18 years, diagnosis of congenital hypothyroidism or secondary immunodeficiency, incomplete thyroid-related data, and inaccessible medical records. All patients were classified according to the International Union of Immunological Societies (IUIS) guidelines [23].

### Data collection

Data were collected retrospectively from patient records. Variables analyzed included demographic characteristics (age, sex, age at symptom onset, and age at IEI diagnosis), IEI phenotype, and results of genetic testing. Thyroid-related parameters included age at AITD diagnosis, thyroid medication use, thyroid functional status (euthyroid, hypothyroid, or hyperthyroid), presence of thyroid autoantibodies (anti-TPO, anti-Tg, and/or TRAb), and thyroid USG features. Information on non-thyroidal autoimmune manifestations and additional immunological data—such as antinuclear antibodies (ANA) positivity, lymphocyte subsets, and B-cell profiles—were also recorded.

### Definitions of thyroid abnormalities

There is currently no universally accepted diagnostic standard for AITD in the context of IEI. In this study, we applied a stepwise definition to balance diagnostic specificity with clinical relevance and to account for the potential passive transfer of autoantibodies after IGRT [21]. For the purposes of analysis, thyroid-related findings were classified into two main categories:

1. Autoimmune Thyroid Disease (AITD): including seropositive AITD and SN-CAT.
2. Non-AITD thyroid abnormalities: including isolated thyroid serology positivity and other non-autoimmune thyroid abnormalities (e.g., solitary thyroid nodule).

Seropositive AITD was defined by the mandatory presence of thyroid autoantibody positivity (anti-TPO and/or anti-Tg and/or TRAb) together with at least one of the following: (1) thyroid USG features compatible with autoimmune thyroiditis (reduced echogenicity, heterogeneity and hypervascularity, as well as the presence of small cysts), (2) persistent biochemical thyroid function test abnormality, or (3) ongoing L-thyroxine treatment [4, 24, 25].

SN-CAT referred to patients who tested negative for all three antibodies but demonstrated USG features typical of autoimmune thyroiditis, with or without abnormal thyroid function tests [6–8].

Isolated thyroid serology was defined as positive thyroid autoantibodies (anti-TPO, anti-Tg, and/or TRAb) in the absence of abnormal thyroid USG features and with normal thyroid function tests.

### Statistical analysis

Statistical analyses were performed using SPSS version 28. Continuous variables were presented as mean ± standard deviation (SD) or median with interquartile range (IQR), depending on distribution. Categorical variables were expressed as frequencies and percentages. For comparisons between groups, the Student’s t-test or Mann–Whitney U test was applied for continuous variables, while the Chi-square or Fisher’s exact test was used for categorical variables. Pearson or Spearman correlation coefficients were calculated to assess the relationship between continuous variables. A *p*-value of less than 0.05 was considered statistically significant.

## Results

### Patient demographics and clinical characteristics

A total of 45 adult patients diagnosed with IEI were included in the study. Of these, 21 (46.7%) were female and 24 (53.3%) were male. The median age of the study population was 34 years (range = 18–70). The median age at symptom onset was 8 years (range = 1–40), and the median age at IEI diagnosis was 25 years (range = 3–64). The median diagnostic delay was calculated as 10 years (range = 1–40). Among those with available data, 9.5% (4/42) were current smokers, and 29.7% (11/37) were actively employed at the time of the study. The median body mass index (BMI) was 23.4 kg/m² (range = 19.5–27.5). A family history of IEI was noted in 4/42 patients (9.5%), and parental consanguinity in 15/42 patients (35.7%).

**Fig. 1:**
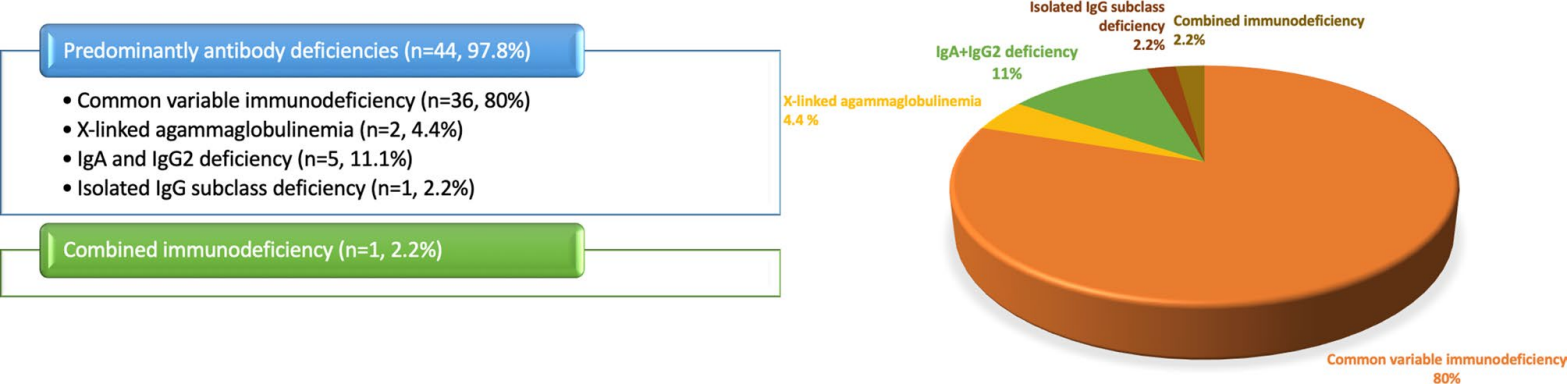
Distribution of inborn errors of immunity phenotypes in the study cohort, classified according to the International Union of Immunological Societies criteria. Abbreviation: Ig, Immunoglobulin

According to the IUIS classification, the vast majority (97.8%, *n* = 44) had predominantly antibody deficiencies, while one patient (2.2%) was classified with combined immunodeficiency (Fig. 1). Immunoglobulin replacement therapy was administered to 41 patients (91.1%), of whom 36 (80%) received intravenous immunoglobulin (IVIG) and 5 (11.1%) subcutaneous immunoglobulin (SCIG). Additionally, 24 patients (53.3%) were on antimicrobial prophylaxis. Among the study cohort, 26 patients (57.8%) had negative thyroid serology and normal thyroid function tests; of these, 15 underwent thyroid USG, all of which were reported as normal. Isolated thyroid serology, in the absence of abnormal USG features or thyroid dysfunction, was observed in 11 patients (24.4%). Six patients (13.3%) fulfilled the criteria for seropositive AITD, while one patient (2.2%) was classified as having SN-CAT. In addition, one patient (2.2%) presented with a solitary thyroid nodule without any evidence of AITD.

Additionally, four patients (8.8%) had other endocrine disorders, including non-thyroidal endocrinopathies such as type 2 diabetes mellitus and growth retardation. ANA were negative in all patients.

### Characteristics of patients with AITD in the study cohort

In the study cohort, seven patients (15.5%) were diagnosed with AITD. Of these, one patient was classified as having SN-CAT, and six as having seropositive AITD. All patients with seropositive AITD were diagnosed with HT, and no cases of GD were identified. All patients with AITD tested negative for TRAb. Anti-TPO antibodies were positive in 85.7% (6/7) of cases, while anti-Tg antibodies were positive in 60.0% (3/5) of those tested. Three patients (42.8%) had concomitant extra-thyroid autoimmune diseases, two of whom had immune thrombocytopenic purpura (ITP).

**Fig. 2:**
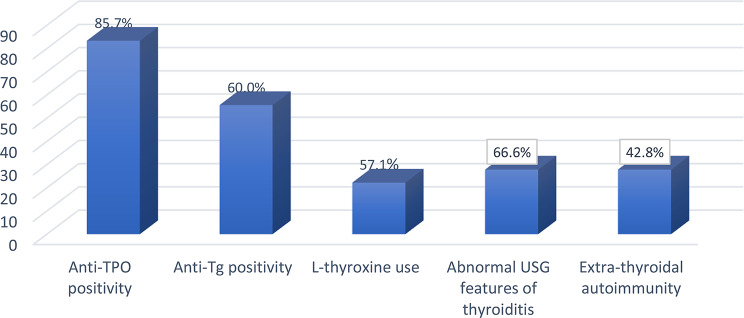
Clinical, serological, and ultrasonographic characteristics of autoimmune thyroid diseases patients (*n* = 7). Abbreviations: Anti-TPO, anti-thyroid peroxidase; Anti-Tg, anti-thyroglobulin; USG, ultrasonography

The proportions of patients with positive thyroid autoantibodies, ultrasonographic findings compatible with autoimmune thyroiditis, and concomitant extrathyroidal autoimmune diseases among those with AITD are presented in Fig. 2. All patients with AITD were euthyroid at the time of assessment; however, four patients (57.1%) were receiving L-thyroxine therapy. Detailed clinical, serological, and radiological data for patients with AITD are provided in Table 1.

### Characteristics of patients with non-AITD thyroid abnormalities in the study cohort

In the study cohort, 12 patients (26.6%) were classified as having non-AITD thyroid abnormalities. Of these, one patient had a solitary thyroid nodule. Thyroid USG in this patient showed normal thyroid size and homogeneous parenchymal echotexture, with a 16 mm peripherally calcified nodule in the right lobe, a mixed-type nodule with a 3.5 × 3 mm iso-hypoechoic solid component in the lower pole of the right lobe, and a 3.5 mm septated cyst in the mid-portion of the left lobe. Fine-needle aspiration biopsy (FNAB) of the peripherally calcified nodule had yielded an indeterminate cytological result, and a second FNAB was planned; the patient was undergoing further diagnostic work-up at the time of data collection. The remaining 11 patients were classified as having isolated thyroid serology, as defined above; all were receiving IGRT, and thyroid antibody testing had been performed 21–28 days after their most recent IGRT infusion. Detailed clinical, serological, and radiological data for patients with isolated thyroid serology are provided in Table 1.

**Table 1:** Characteristics of patients diagnosed with AITD and isolated thyroid serology

Case	Age* /Sex	IEI subtype	AITD definition	Extra-thyroidal autoimmunity	Thyroid autoantibodies	Thyroid USG features	AITD treatment
1	50 /M	CVID	SN-CAT	ITP	Anti-TPO (-) Anti-Tg (-)	Nodules on thyroiditis background	
2	34 /M	CVID	Isolated thyroid serology	ITP	Anti-TPO (+) Anti-Tg (+)	Normal	
3	29 /F	CID	Isolated thyroid serology	ITP	Anti-TPO (+)	Normal	
4	61 /F	CVID	Seropositive AITD	ITP	Anti-TPO (+) Anti-Tg (+)	Post-thyroidectomy	L-thyroxine
5	60 /F	CVID	Isolated thyroid serology	No	Anti-TPO (+)	NA	
6	18 /M	CVID	Isolated thyroid serology	No	Anti-TPO (-) Anti-Tg (+)	Normal	
7	39 /F	CVID	Seropositive AITD	No	Anti-TPO (+) Anti-Tg (-)	Consistent with thyroiditis	
8	70 /F	CVID	Seropositive AITD	No	Anti-TPO (+) Anti-Tg (+)	Nodules on thyroiditis + FNAB	L-thyroxine
9	33 /M	CVID	Isolated thyroid serology	No	Anti-TPO (+) Anti-Tg (+)	Normal	
10	41 /F	CVID	Isolated thyroid serology	No	Anti-TPO (+) Anti-Tg (+)	Normal	
11	21 /M	CVID	Isolated thyroid serology	No	Anti-TPO (-) Anti-Tg (+)	NA	
12	34 /F	CVID	Seropositive AITD	No	Anti-TPO (+)	NA	L-thyroxine
13	26 /M	CVID	Isolated thyroid serology	No	Anti-TPO (+)	NA	
14	41 /M	CVID	Isolated thyroid serology	No	Anti-TPO (+) Anti-Tg (+)	NA	
15	44 /F	CVID	Seropositive AITD	No	Anti-TPO (+) Anti-Tg (+)	NA	L-thyroxine
16	47 /F	CVID	Isolated thyroid serology	No	Anti-TPO (+)	NA	
17	23 /M	X-LA	Isolated thyroid serology	No	Anti-TPO (+) Anti-Tg (+)	NA	
18	46 /M	CVID	Seropositive AITD	Celiac, vitiligo, alopecia	Anti-TPO (+)	Consistent with thyroiditis	

Abbreviations: *, years; AITD, autoimmune thyroid disease; IEI, inborn errors of immunity; USG, ultrasonography; SN-CAT: Seronegative chronic autoimmune thyroiditis; FNAB, fine-needle aspiration biopsy; Anti-TPO, anti-thyroid peroxidase antibody; Anti-Tg, anti-thyroglobulin antibody; HT, Hashimoto’s thyroiditis; CVID, Common variable immunodeficiency; X-LA, X-linked agammaglobulinemia; CID, Combined immunodeficiency; ITP, Immune thrombocytopenic purpura; RA, Rheumatoid arthritis; NA, Not available

### Comparison of clinical and laboratory characteristics between AITD and Non-AITD patients

There were no statistically significant differences between patients with and without AITD in terms of sex distribution (*p* = 0.225), clinical phenotype (*p* = 0.722), presence of bronchiectasis (*p* = 1.000), infectious complications (*p* = 1.000), extra-thyroidal autoimmune diseases (*p* = 0.394), extra-thyroidal endocrine comorbidities (*p* = 0.505), or hematological comorbidities (*p* = 1.000). Among lymphocyte subsets, only the proportion of CD16⁺56⁺ natural killer (NK) cells differed significantly between groups, with patients with AITD exhibiting a markedly higher percentage than individuals without AITD (16.34% ± 14.53% vs. 7.63% ± 5.20%, respectively; *p* = 0.010). No significant differences were observed for CD19⁺ B cells, CD3⁺CD4⁺ T cells, or CD3⁺CD8⁺ T cells (all *p* > 0.05). For CD45RA/RO subpopulations, the small sample size precluded reliable inference.

## Discussion

In this study, we evaluated the prevalence, clinical characteristics, and immunological profile of AITD in adult patients with IEI. AITD was identified in 15.5% of cases, of which six were classified as HT and one as SN-CAT. Five patients (71.4%) were female. Extra-thyroidal autoimmune comorbidities were present in 42.8% of cases. Four patients were receiving L-thyroxine therapy, and all AITD cases were euthyroid at the time of assessment. Immunological analysis revealed significantly higher levels of CD16⁺56⁺ NK cells in patients with AITD, a finding that may contribute to the understanding of thyroid autoimmunity pathogenesis in the context of IEI.

AITDs, particularly HT, affect 2–5% of the general population in Western countries, with prevalence varying by geography, socioeconomic status, age, and sex, and occurring more frequently in females [12, 13, 26]. These disorders are also among the most frequently reported autoimmune manifestations in IEI cohorts, especially in subtypes such as common variable immunodeficiency (CVID), IPEX syndrome (immunodysregulation, polyendocrinopathy, enteropathy, X-linked syndrome), and Hyper-IgM syndrome (HIGM) [27, 28]. For instance, a study involving 92 adult patients with heterogeneous IEI subtypes reported an AITD prevalence of 14% [19]. Another investigation focusing on CVID patients found that 29% exhibited autoimmunity, with AITD being among the most frequently identified conditions [29]. Similarly, a large pediatric cohort of 1,036 IEI patients revealed autoimmune or inflammatory features in 10% of individuals, among whom AITD was most commonly reported manifestation (25.3%) [30].

In our cohort, which consisted predominantly of patients with CVID (90%), the prevalence of AITD was 15.5%. This rate is markedly higher than that reported in the general population and is consistent with the prevalence documented in some previous IEI studies. We believe that discrepancies in prevalence rates across IEI studies may be attributable to differences in the age distribution of study populations, the spectrum of included IEI subtypes, and variations in the diagnostic criteria applied. A recent systematic review supports this view, noting substantial geographic and methodological variation in AITD prevalence [26]. These discrepancies underscore the heterogeneity of autoimmune features in IEI and the importance of context when interpreting findings across studies. In this light, the high rate of AITD observed here reinforces its relevance as a common noninfectious complication in IEI, particularly in CVID-dominant populations. Given its often subclinical course and systemic effects, including effects on metabolism, cognitive, and behavioral impacts, we believe that routine thyroid evaluation is of significant value in long-term IEI care.

Sex-specific patterns in AITD are noteworthy. In our cohort, although there was no statistically significant difference in sex distribution between patients with and without AITD, 71.4% of AITD cases were female. This finding aligns with the well-established female predominance reported in the general population. However, it contrasts with two previous studies from Türkiye involving IEI patients, neither of which identified significant sex-based disparities [18, 19]. One of these studies suggested that the male predominance within their cohort may have influenced the observed outcome. Thus, while our finding supports the established female predominance seen in the general population, it diverges from prior national data on sex distribution in IEI. This discrepancy may stem from methodological differences between studies or from the heterogeneous nature of IEI, which could modify the typical sex-related susceptibility pattern associated with AITD.

In addition to demographic and prevalence characteristics, diagnostic considerations, including the role of thyroid autoantibodies, are particularly relevant in the context of IEI. HT is typically diagnosed through a combination of clinical findings, serological markers, and, when available, histopathological evidence. Anti-TPO antibodies are regarded as the most specific marker, detectable in approximately 95% of patients, while anti-Tg antibodies are found in 60–80% of cases [25]. Thyroid autoantibodies may also be present in asymptomatic individuals, reflecting a potentially high burden of subclinical disease [31, 32].

In IEI, however, the diagnostic reliability of autoantibody testing is limited by two opposing factors. On the one hand, several studies have reported that impaired humoral responses in this population may lead to false-negative results, with autoantibodies remaining undetectable despite underlying autoimmune pathology [14, 20]. On the other hand, in patients receiving IGRT, false-positive results may arise from the passive transfer of thyroid autoantibodies [21]. Reports of thyroid antibody detection are particularly common after high-dose IVIG therapy, although such regimens were not applied in our cohort [33, 34]. In our cohort, although all patients were on IGRT, only 11 (24.4%) demonstrated isolated seropositivity, while the majority (62.3%) remained negative, indicating that seropositivity cannot be uniformly attributed to passive transfer. To address this, we classified patients with isolated antibody positivity but completely normal thyroid USG and thyroid function tests as non-AITD. Importantly, prior work has shown that the likelihood of clinically significant AITD is very low when thyroid ultrasound findings remain persistently normal, with Pedersen et al. reporting a negative predictive value of 93% for excluding AITD in such cases [35]. Nevertheless, given evidence that isolated antibody positivity may represent an early stage of autoimmunity, this subgroup warrants close follow-up. Thyroid USG thus provides additional diagnostic value, as it can identify characteristic parenchymal heterogeneity and nodularity even in seronegative individuals [6, 7]. Given the limitations of serological markers in IEI, we believe that, alongside antibody testing, the integration of imaging modalities and thyroid function monitoring provides a more reliable framework for accurate diagnosis in this population [25].

Our findings suggest that patients with IEI require careful, individualized evaluation for AITD. In our cohort, some patients were diagnosed based on USG features despite negative autoantibodies, while others showed seropositivity without clinical or USG evidence, highlighting the complexity of interpreting thyroid autoantibodies in this population. Considering the potential consequences of thyroid dysfunction, the presence of seronegative cases, and the risk that isolated antibody positivity may represent an early stage of disease, cautious interpretation of test results is warranted. In this context, longitudinal monitoring of thyroid function and structure appears particularly valuable, even before overt clinical symptoms emerge.

Patients with AITD in our cohort demonstrated increased frequencies of CD16⁺56⁺ NK cells, which are implicated in antibody-dependent cellular cytotoxicity and proinflammatory cytokine production [36]. Other alterations reported in the literature, such as expansion of CD8⁺CD45RA⁺ T cells, a terminally differentiated effector subset linked to chronic immune activation and autoimmune pathology, and elevated IgG2 levels, considered a marker of Th1-associated antibody responses and impaired immune regulation, were not observed in our sample, possibly due to the limited cohort size [37–39]. Taken together, these observations, particularly the NK cell expansion, support the notion of an activated and dysregulated immune environment potentially contributing to thyroid autoimmunity in IEI. Distinct immunological alterations observed in AITD patients within our IEI cohort may therefore provide insight into thyroid-specific autoimmunity in this context, and could be explored in future studies to better delineate underlying mechanisms. Future studies incorporating larger cohorts and longitudinal immunophenotyping will be essential to validate these findings and to clarify how dynamic changes in immune cell subsets shape thyroid autoimmunity in this setting.

This study has several limitations. First, its retrospective and single-center design may limit generalizability. Second, the relatively small sample size, particularly within certain IEI subgroups, reduces the statistical power for subgroup analyses. Third, the absence of longitudinal follow-up precluded assessment of temporal changes in thyroid function, antibody profiles, and disease progression. Fourth, genetic analyses were restricted, limiting insights into genotype–phenotype correlations.

In terms of diagnostic evaluation, thyroid USG was not available for all patients; notably, imaging data were missing in 8 individuals with non-AITD thyroid abnormalities and in some seronegative cases, potentially reducing sensitivity for case detection. Furthermore, impaired humoral responses in IEI constrain the reliability of autoantibody testing, while IGRT may introduce confounding through passive antibody transfer. To address this, we applied a stepwise definition of AITD and did not accept isolated seropositivity in IGRT recipients as sufficient for classification, a strategy intended to minimize misclassification and improve diagnostic specificity.

Finally, Selective IgA deficiency was excluded as it represents an IEI subtype with distinct clinical features and a high prevalence of autoimmunity, particularly thyroiditis [40, 41]. The aim of this exclusion, together with consideration of heterogeneity between IGRT recipients and non-recipients, was to reduce confounding and improve the internal consistency of the study population.

Taken together, these limitations not only contextualize the interpretation of our findings but also emphasize key areas for clinical practice: the need for systematic thyroid function testing, USG, and careful interpretation of serology in IEI patients, even in the absence of overt symptoms.

## Conclusions

Our study demonstrates a notable prevalence of autoimmune thyroid disease among adults with IEI, often in the absence of overt clinical signs. Seronegative presentations and distinct immunological patterns highlight the risk of underrecognition without systematic screening. Because autoantibody testing in IEI is limited by impaired humoral responses and the confounding effect of IGRT, complementary tools such as thyroid ultrasound and longitudinal follow-up are essential. These findings underscore the need for systematic thyroid evaluation in IEI and highlight the importance of conducting larger, prospective studies in genetically diverse cohorts to further elucidate underlying mechanisms and optimize diagnostic strategies.

## Data Availability

The authors declare that they have followed the protocols of their work center for the publication of patient data in this study. The datasets generated and/or analysed during the current study are not publicly available due [The data are not publicly available due to privacy or ethical restrictions] but are available from the corresponding author on reasonable request.
